# Application of Highly Flexible Adaptive Image Receive Coil for Lung MR Imaging Using Zero TE Sequence: Comparison with Conventional Anterior Array Coil

**DOI:** 10.3390/diagnostics12010148

**Published:** 2022-01-08

**Authors:** Kyungsoo Bae, Kyung Nyeo Jeon, Moon Jung Hwang, Yunsub Jung, Joonsung Lee

**Affiliations:** 1 Department of Radiology, Institute of Health Sciences, School of Medicine, Gyeongsang National University, Jinju 52727, Korea; ksbae@gnu.ac.kr; 2 Department of Radiology, Gyeongsang National University Changwon Hospital, Changwon 51472, Korea; 3 General Electronics (GE) Healthcare Korea, Seoul 04637, Korea; moonjung.hwang@ge.com (M.J.H.); Yunsub.Jung@ge.com (Y.J.); Joonsung.Lee@ge.com (J.L.)

**Keywords:** lung, magnetic resonance imaging, zero-echo-time imaging, adaptive image receive coil, ultrashort T2

## Abstract

(1) Background: Highly flexible adaptive image receive (AIR) coil has become available for clinical use. The present study aimed to evaluate the performance of AIR anterior array coil in lung MR imaging using a zero echo time (ZTE) sequence compared with conventional anterior array (CAA) coil. (2) Methods: Sixty-six patients who underwent lung MR imaging using both AIR coil (ZTE-AIR) and CAA coil (ZTE-CAA) were enrolled. Image quality of ZTE-AIR and ZTE-CAA was quantified by calculating blur metric value, signal-to-noise ratio (SNR), and contrast-to-noise ratio (CNR) of lung parenchyma. Image quality was qualitatively assessed by two independent radiologists. Lesion detection capabilities for lung nodules and emphysema and/or lung cysts were evaluated. Patients’ comfort levels during examinations were assessed. (3) Results: SNR and CNR of lung parenchyma were higher (both *p* < 0.001) in ZTE-AIR than in ZTE-CAA. Image sharpness was superior in ZTE-AIR (*p* < 0.001). Subjective image quality assessed by two independent readers was superior (all *p* < 0.05) in ZTE-AIR. AIR coil was preferred by 64 of 66 patients. ZTE-AIR showed higher (all *p* < 0.05) sensitivity for sub-centimeter nodules than ZTE-CAA by both readers. ZTE-AIR showed higher (all *p* < 0.05) sensitivity and accuracy for detecting emphysema and/or cysts than ZTE-CAA by both readers. (4) Conclusions: The use of highly flexible AIR coil in ZTE lung MR imaging can improve image quality and patient comfort. Application of AIR coil in parenchymal imaging has potential for improving delineation of low-density parenchymal lesions and tiny nodules.

## 1. Introduction

Magnetic resonance (MR) is an attractive imaging tool that can provide both morphological and functional information of objects without ionizing radiation. However, MR imaging of the lung has been challenging because of low proton density in the lung and rapid signal decay caused by susceptibility effects at the air–tissue interface [1]. Recently, sequences using very short echo time (TE) such as ultrashort echo (UTE) and zero echo (ZTE) sequences have gained attention due to their capability of imaging ultrashort T2* tissues such as lung, bone, and ligament [2]. It has been shown that integration of UTE or ZTE in routine chest MR protocol can provide high-resolution structural information of the lung, potentially eliminating the need for CT [3,4,5]. Minimal gradient switching in a ZTE pulse sequence can drastically decrease patient annoyance due to acoustic noise [6]. A recent direction of research involves respiratory gating of these sequence to generate 4D images for assessing respiratory motion and regional ventilation [7,8].

Because of a relatively long scan time and the vulnerability to respiratory motion artifacts in lung MR imaging, patients’ comfort is a very important factor for high-quality imaging [1,9]. Owing to confined spatial environment in an MR machine, some patients become nervous and poorly cooperative to respiratory instructions during examination. Conventional coil elements are fixed in rigid housing to prevent damage with repeated use. The weight and pressure of rigid coil arrays on a patient’s chest can cause discomfort and affect the patient’s breathing patterns [9].

Many efforts have been made in the development and elaboration of flexible MR coils [10,11,12]. Recently, adaptive image receive (AIR) coil array has become available for clinical use [13]. This coil array comprises of flexible coil elements together with highly miniaturized electronic components. These elements are then enclosed in a lightweight, flame-retardant padded textile. Owing to its flexibility and light weight, AIR coil is expected to maintain a close patient fit and offer comfortable examination. However, limited information is available about the clinical performance of AIR coil. The feasibility of prototype balaclava AIR coil for whole-brain imaging has been reported [14]. AIR coil in a blanket type has been introduced for various body parts including spine, chest, abdomen, and extremities [15]. The feasibility and performance of AIR coil in body imaging has not yet been studied. Therefore, the purpose of this study was to evaluate the performance of a 30-channel AIR coil in structural lung MR imaging using ZTE sequence from a clinical perspective in comparison with 16-channel conventional anterior array (CAA) coil commonly used for body imaging in practice.

## 2. Materials and Methods

### 2.1. Subjects

Our Institutional Review Board approved this study. Written informed consent was obtained from all patients. From May 2019 to March 2021, 66 consecutive adult patients underwent chest MRI for further evaluation of thoracic lesions detected on chest CTs obtained using a multidetector scanner. Indication of chest MR was staging of lung cancer (*n* = 31) and esophageal cancer (*n* = 2), differential diagnosis of lung nodule/mass (*n* = 7), evaluation of pleural (*n* = 1), mediastinal (*n* = 23), and chest wall lesion (*n* = 2). There were 50 males and 16 females with a mean age of 61.2 years (range, 19–88 years). Body mass index of patients ranged from 16.3 to 35.5 (mean, 23.6).

### 2.2. Image Acquisition

MR images were obtained using a commercial 3T scanner (Signa Architect, GE Healthcare, Milwaukee, WI, USA). ZTE imaging is performed as part of routine chest MRI protocol at our institution (Gyeongsang University Changwon Hospital, Changwon, Korea). Two sets of ZTE lung MR imaging were obtained using 16-channel CAA (ZTE-CAA) and 30-channel AIR coils (ZTE-AIR), respectively, combined with a 40-channel posterior array coil. The order of the two ZTE scanning was random for each patient.

Scans were performed during quiet breathing. Signals of respiratory bellows wrapped around the patient’s upper abdomen were used as surrogates of respiratory motion. Data were prospectively acquired when the position of the diaphragm was within an acceptance window during approximately one-third of the end-expiration phase. Coronal images with isotropic resolution of 1.5 mm were obtained. Original coronal image data were reformatted into axial images. ZTE scan parameters in both scans were as follows: repetition time, 393~503 ms; echo time (ΔT), 16 μs; flip angle, 2°; No. of spokes per segment, 256; field of view, 384 × 384 mm^2^; receiver bandwidth, ±31.25 kHz; respiration trigger window, 30% of respiratory cycle; and mean scan time, 137 s (127–148 s).

### 2.3. Quantitative Analysis

To compare quantitative image qualities between ZTE-AIR and ZTE-CAA, one radiologist with 7 years of experience who was blind to the purpose of this study drew circular regions of interest (ROIs) in both lungs and background air for all patients. When placing ROI in the lung, vascular markings and fissures were avoided. Measurements were performed in three different areas of each lung and background air. The mean of measurements was considered as the representative value for each structure or parameter. Standard deviation (SD) of the intensity measured in background air was considered as image noise. Signal-to-noise ratio (SNR) and contrast-to-noise ratio (CNR) of lung parenchymal were calculated according to the following equations: SNR = mean signal intensity (SI) of lung/image noise; CNR = (mean SI of lung–mean SI of back-ground air)/image noise.

$$\mathrm{SNR}\left(\mathrm{lung}\right)=\frac{\mathrm{mean}\;\mathrm{SI}\;\mathrm{of}\;\mathrm{lung}}{\mathrm{image}\;\mathrm{noise}}$$

$$\mathrm{CNR}\left(\mathrm{lung}\right)=\frac{\mathrm{mean}\;\mathrm{SI}\;\mathrm{of}\;\mathrm{lung}-\mathrm{mean}\;\mathrm{SI}\;\mathrm{of}\;\mathrm{background}\;\mathrm{air}}{\mathrm{image}\;\mathrm{noise}}$$

Image sharpness of ZTE-AIR and ZTE-CAA was evaluated using blur metric. For blur metric analysis, one radiologist with 7 years of experience marked rectangular ROIs on the trachea of original coronal images (Figure S1). ROIs were drawn in the same location on ZTE-CAA and ZTE-AIR for each patient. One computer scientist then calculated blur metric values from radiologist-marked ROI areas for each image data set. We developed a MATLAB (Mathworks, Natick, MA, USA)-based analysis program and added a blur metric function developed by the original developer to our program [16]. Blur metric, which quantitatively analyzes the sharpness of an image, is a no-reference-based measurement method that can estimate the level of sharpness using the degree of variation for adjacent pixel values [17,18,19]. The calculated value ranged from 0 to 1, with a lower value indicating a sharper image and a higher value indicating a blurrier image.

### 2.4. Qualitative Analysis

Two radiologists with 25 and 26 years of experience, respectively, independently evaluated ZTE-CAA and ZTE-AIR in terms of visualization of normal structures (intrapulmonary vessels, bronchi, and fissures,), degree of noise and artifacts, and overall acceptability. Both coronal and axial images were reviewed. The score of each category was rated using a five-point scale (Table 1).

### 2.5. Lesion Evaluation

To evaluate the capability for parenchymal lesion detection on ZTE images, two board-certified chest radiologists with 24 and 25 years of experience, respectively, reviewed thin-section chest CT scans. Images were evaluated for the presence of nodule (≤3 cm), emphysema, and lung cysts including bullae (>1 cm). The final decision for each patient was determined as consensus by two radiologists. It served as the reference standard for lesion evaluation in this study.

Two readers with 7 and 6 years of experience in chest imaging, respectively, who were blinded to the diagnosis evaluated all ZTE image data sets during two reading sessions within a two-week interval. Two ZTE data sets of the same patient were not provided during the same reading session. For evaluation, lungs were divided into four zones (right upper [RU], right lower [RL], left upper [LU], and left lower [LL] zones) using the bronchus intermedius as reference. Readers were asked to record the presence or absence of nodules, emphysema, and/or lung cysts on each lung zone. When there were multiple nodules in a lung zone, up to three nodules in size order (from the largest to the smallest) were evaluated. Mass, consolidation, and parenchymal distortion were ignored. The diagnostic confidence for lesion was rated using a five-point scale (1, definitely absent; 2, probably absent; 3, suspicious; 4, probably present; 5, definitely present). Scores of 3, 4, and 5 were considered positive for detection. Diagnostic performance for nodule was evaluated per nodule and per zone-by-zone basis. Diagnostic performance for emphysema/bullae was evaluated on a zone-by-zone basis.

### 2.6. Assessment of Patients’ Comfort

After scanning, patients were asked to rate the level of comfort during two ZTE scans on a 5-point scale: score 1, much more comfortable during ZTE-CAA; score 2, slightly more comfortable during ZTE-CAA; score 3, equally comfortable; score 4, slightly more comfortable during with ZTE-AIR; and score 5, much more comfortable during ZTE-AIR.

### 2.7. Statistical Analysis

The Wilcoxon signed-rank test was used to compare differences in SNR and CNR of lung parenchyma and image sharpness between ZTE-CAA and ZTE-AIR. Subjective image quality between ZTE-CAA and ZTE-AIR was also compared using the Wilcoxon signed-rank test. McNemar’s test was used to compare diagnostic performances of ZTE-CAA and ZTE-AIR for lung nodule and emphysema. The effect size (also known as strength of association) was estimated using eta squared (η^2^). Effect sizes were interpreted as follows: 0.01 ≤ η^2^ < 0.06, small effect; 0.06 ≤ η^2^ < 0.14, moderate effect; and η^2^ ≥ 0.14, large effect.

Inter-reader agreements for qualitative assessment and lesion evaluation were determined by calculating the weighted kappa coefficient. The weighted kappa value was interpreted as follows: 0.20 or less, poor; 0.21–0.40, fair; 0.41–0.60, moderate; 0.61–0.80, substantial; and 0.81 or greater, almost perfect agreement. All statistical analyses were performed using SPSS package version 24.0 (SPSS Inc., Chicago, IL, USA). *p* values of less than 0.05 indicated statistical significance.

## 3. Results

ZTE lung imaging was successfully performed for all patients without any adverse events.

### 3.1. Quantitative Evaluation

Both SNR and CNR of lung parenchyma were significantly (both *p* < 0.001) higher in ZTE-AIR than in ZTE-CAA. Blur matric value of ZTE-AIR was significantly (*p* < 0.001) lower, indicating better image sharpness than ZTE-CAA (Table 2).

### 3.2. Qualitative Evaluation

Subjective image qualities assessed by two independent readers regarding visualization of intrapulmonary structures (vessels, airway, and fissures) were significantly higher (all *p* < 0.05) in ZTE-AIR (Table 3). Scores for image noise/artifacts and overall acceptability were superior in ZTE-AIR (all *p* < 0.001) to those in ZTE-CAA.

Inter-reader agreements were substantial in evaluations of intrapulmonary vessels (κ = 0.748 in AIR, κ = 0.824 in CAA), fissures (κ = 0.773 in AIR, κ = 0.779 in CAA), image noise/artifacts (κ = 0.775 in AIR, κ = 0.756 in CAA), and overall acceptability (κ = 0.781 in AIR, κ = 0.77 in CAA) in both ZTE datasets. Inter-reader agreements for bronchi (κ = 0.588 in AIR, κ = 0.423 in CAA) were moderate in both ZTE datasets (Table S1).

### 3.3. Lesion Detection

There were 80 pulmonary nodules in 54 lung zones of 38 patients. Sizes of nodules ranged from 3 mm to 30 mm (mean, 10.6 mm). A total of 51 nodules were less than 1 cm in diameter. There were emphysema and/or bullae in 46 lung zones of 22 patients.

**Figure 1:**
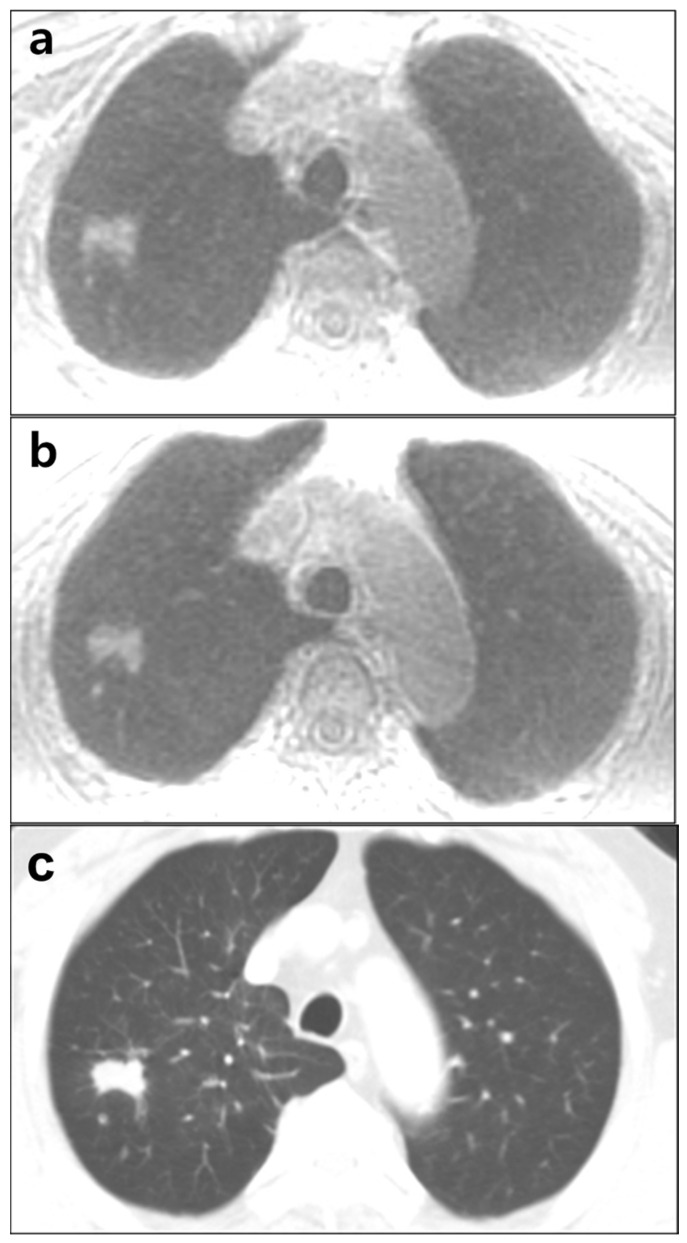
A 61-year-old male with lung cancer in the right upper lobe. A 24 mm-sized primary nodule and a tiny satellite nodule in the right upper lobe in ZTE-CAA (**a**) and ZTE-AIR (**b**) are shown. Chest CT image (**c**) is presented as reference. ZTE-CAA, zero echo time lung MR image using conventional anterior array coil; ZTE-AIR, zero echo time lung MR image using adaptive image receive coil.

**Figure 2:**
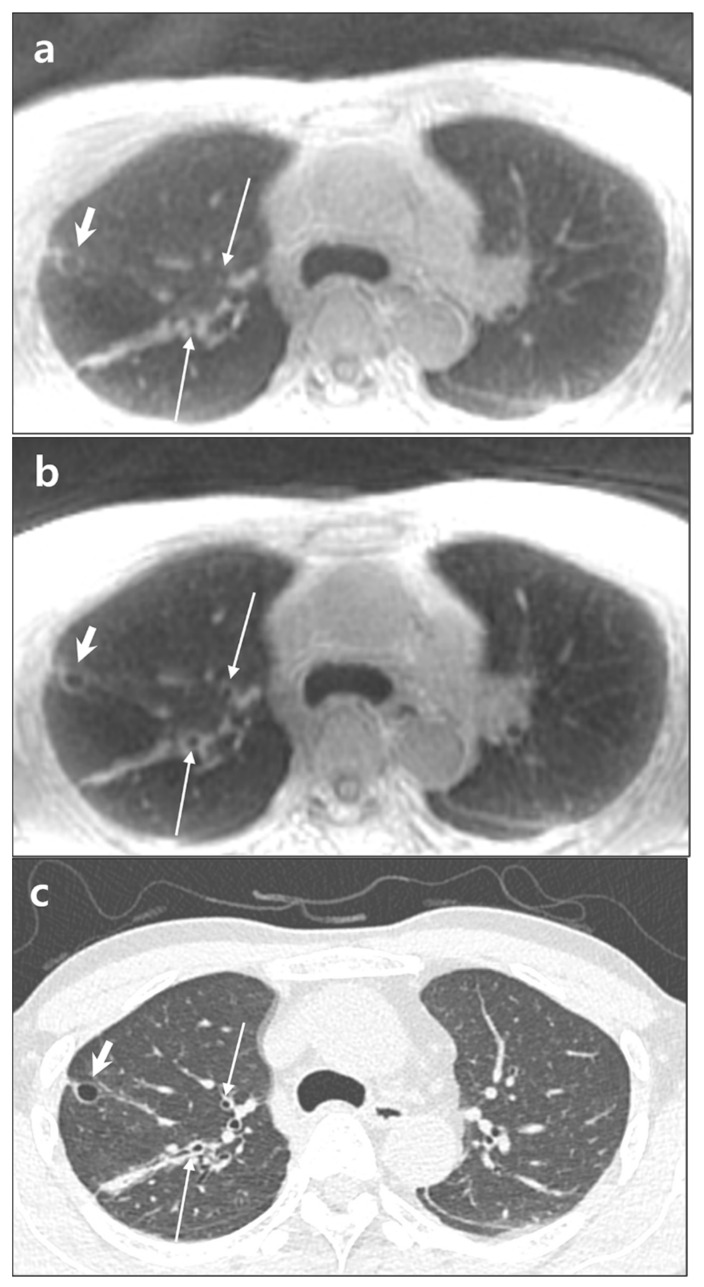
A 55-year-old male with lung cyst in the right upper lobe. Note signal difference between lung parenchyma and cyst (short arrows) and visualization of peripheral bronchi (thin arrows) in ZTE-CAA (**a**) and ZTE-AIR (**b**). Chest CT image (**c**) is presented as reference. ZTE-CAA, zero echo time lung MR image using conventional anterior array coil; ZTE-AIR, zero echo time lung MR image using adaptive image receive coil.

**Figure 3:**
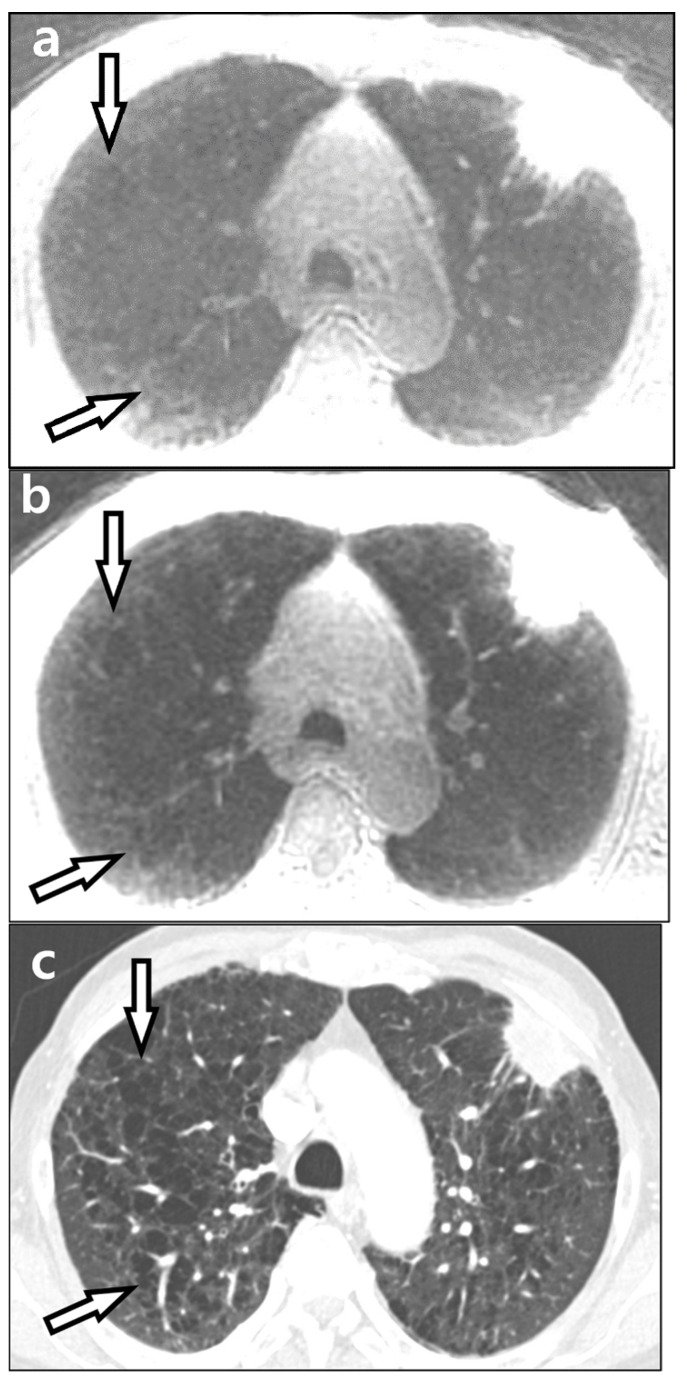
An 83-year-old male with lung cancer. A lobulated mass in the left upper lobe is well-defined in both ZTE-CAA (**a**) and ZTE-AIR (**b**). However, the presence of emphysema is more clearly defined in ZTE-AIR. CT image is presented for comparison (**c**). ZTE-CAA, zero echo time lung MR image using conventional anterior array coil; ZTE-AIR, zero echo time lung MR image using adaptive image receive coil.

Diagnostic performances of ZTE-CAA and ZTE-AIR in detecting nodules on zone-by-zone and nodule-base analyses by both readers were not significant different (Table 4). In subgroup analysis according to nodule size, ZTE-AIR showed significantly higher (both *p* < 0.05) sensitivity than ZTE-CAA for sub-centimeter nodules by both readers (Table 5, Figure 1). Sensitivity and accuracy for detecting emphysema and/or cysts were significantly (all *p* < 0.05) higher for ZTE-AIR than ZTE-CAA by both readers (Figure 2 and Figure 3). Specificity for detecting emphysema was not significantly different between ZTE-AIR than ZTE-CAA for both readers. Inter-reader agreements in evaluations of nodules (κ = 0.75 in AIR, κ = 0.616 in CAA) and emphysema and/or lung cysts (κ = 0.685 in AIR, κ = 0.7 in CAA) were substantial in both ZTE datasets (Table S1).

Inter-reader agreement on nodule-wise evaluation was moderate (κ = 0.574 for ZTE-AIR, κ = 0.421 for ZTE-CAA) for pulmonary nodules. Inter-reader agreement was substantial for emphysema and/or cysts (κ = 0.754 for ZTE-AIR, κ = 0.751 for ZTE-CAA).

### 3.4. Patients’ Comfort

Of 66 patients, 64 responded that AIR coil was slightly (*n* = 26) or much more (*n* = 38) comfortable than CAA coil (Table S2). The other two patients responded that both coils were equal.

## 4. Discussion

By adopting densely overlapped thin wire loops with a special conductive material named a INCA conductor instead of conventional copper loops, AIR coil has gained lightness and flexibility [13,14]. Conventional coil arrays are fixed in rigid housing to avoid damage and maintain critical overlap between loops for delivering the best SNR. With an INCA conductor connected to a printed circuit board module, AIR coil was designed to reduce coil–coil mutual inductance and coupling between adjacent loops, achieving high decoupling between coil elements and relaxing coil overlap requirements [13,14]. The major interest of this work was to evaluate how such flexibility and interaction between closely overlapped coil elements of 30-channel AIR Anterior Array coil benefit the image quality in lung imaging, combined with a 40-channel posterior array coil.

In the present study, lung parenchyma was 58% higher SNR in ZTE-AIR than ZTE-CAA. CNR of lung parenchyma with background air as reference was also 80% higher in ZTE-AIR. These results reflect higher sensitivity and lower noise level with AIR coil, which might be attributed to both higher channel counts and more compact fit of the coil to patients’ bodies. By conforming to patient anatomy and thereby closely fitting the coil to the object, AIR coil is likely to capture more signal and less noise than CAA coil [20].

Image sharpness measured using blur metric on the trachea was superior in ZTE-AIR than in ZTE-CAA. Owing to lower image noise and higher signal in original ZTE-AIR, intensity variations in the trachea might appear larger in ZTE-AIR, presenting lower blur metric value (i.e., sharper image) than in ZTE-CAA.

With their exceptional capability in imaging ultrashort T2* structures, ultrashort TE sequences including ZTE and UTE showed excellent performances in detecting pulmonary nodules [21,22,23,24]. In the present study, subjective image qualities regarding visualization of intrapulmonary structures such as pulmonary vessels, bronchi, and fissures; degree of image noise/artifacts; and overall acceptability were all superior in ZTE- AIR. Nevertheless, diagnostic performances for lung nodule detection were not different between ZTE-AIR and ZTE-CAA both on per-lung zone and per-nodule base analyses. However, when nodules were subcategorized according to their sizes, ZTE-AIR showed superior sensitivity for sub-centimeter nodules to ZTE-CAA by both readers.

Radiologists’ detection sensitivity for emphysema and/or cysts appeared better in ZTE-AIR than in ZTE-CAA. Pulmonary pathological conditions with low density such as emphysema or bronchiectasis are the most challenging part in lung MR imaging [25,26,27,28]. According to Ohno et al., diagnostic capability of UTE for emphysema/bullae and bronchiectasis was inferior to standard/low-dose CT, while diagnostic capability of UTE for dense parenchymal lesions such as pulmonary nodules and ground-glass opacity was comparable to that of standard/low-dose CT [26]. In the present study, higher CNR of lung parenchyma and lower artifacts in ZTE-AIR could explain its better detectability of area with parenchymal loss, i.e., emphysema or cysts. In addition to functional imaging, improved structural imaging may also contribute to expansion of lung MR in advanced diagnosis and follow-up of chronic lung disease such as chronic obstructive pulmonary disease, interstitial lung disease, and cystic fibrosis [29,30].

In the present study, AIR coil was preferred by most (97%) patients. No patient preferred CAA coil to AIR coil. AIR coil was fit to all patients regardless of their body size or shape. AIR coil did not complicate the placement of medical devices including respiratory bellows.

This study has some limitations. First, we compared performances of AIR coil and CAA coil in a single lung MR sequence. Considering scan time in clinical settings, we preferentially tested the performance of AIR coil in high-resolution structural imaging using ZTE pulse sequence. Second, we could not test the diagnostic performance using various types of lung nodules other than solid nodules because we only had a limited number of cases. Third, better image quality and lesion detectability in ZTE-AIR might not be solely attributable to performance of the coil per se. Since respiratory movement has a great impact on lung MR image quality, the difference in patients’ respiratory cooperation between the two scans could also affect image quality. Fourth, although we used the same scan parameters and protocols, minimal differences between the two scans could affect quantitative evaluation. Last, the geometry of two coils, including number of coil elements, was not equivalent. AIR coil is a new device developed to improve image quality and applicability of MRI. Since our study focused on clinical implication of new coil, the performance of AIR coil was assessed via comparison with currently used coil in practice.

In conclusion, AIR coil offered greater patient comfort and better image quality than CAA coil on high-spatial-resolution ZTE lung MR imaging. Higher SNR and CNR of lung parenchyma on ZTE-AIR show potential for improved diagnosis of tiny nodules and low-density parenchymal lesions such as emphysema.

## Figures and Tables

**Table 1:** Qualitative scoring system of ZTE lung MR images.

**Visualization of intrapulmonary vessels**
1, indistinguishable segmental vessels
2, blurred visualization of segmental vessels
3, clear visualization of segmental vessels
4, visualization of subsegmental vessels
5, visualization of sub-subsegmental vessels
**Visualization of the bronchi**
1, indistinguishable lobar bronchus
2, visualization of lobar bronchus
3, visualization of segmental bronchus
4, visualization of subsegmental bronchus
5, visualization of sub-subsegmental bronchus
**Visualization of fissures**
1, no visualization
2, partial visualization of one fissure
3, partial visualization of two fissures
4, visualization of near whole course of one fissure
5, visualization of near whole course of two fissures
**Noise and artifacts**
1, unacceptable noise/artifacts
2, above-average noise/artifacts
3, average and acceptable noise/artifacts
4, less-than average noise/artifacts
5, minimum or nothing
**Overall acceptability**
1, unacceptable
2, suboptimal
3, satisfactory
4, good
5, excellent

**Table 2:** Comparison of signal-to-noise ratio (SNR), contrast-to-noise ratio (CNR), and image sharpness between ZTE-CAA and ZTE-AIR.

	ZTE-CAA	ZTE-AIR	*p*-Value	Effect Size
**SNR**	7.47 ± 2.81	11.79 ± 5.75	<0.001	0.368
**CNR**	1.17 ± 1.25	2.10 ± 1.84	<0.001	0.147
Image sharpness *	0.49 ± 0.37 *	0.44 ± 0.32 *	<0.001	0.378

* Expressed as blur metric values, with a lower value indicating a sharper image. A *p*-value < 0.05 was considered statistically significant. ZTE-CAA, zero echo time lung MR imaging using conventional anterior array coil; ZTE-AIR, zero echo time lung MR imaging using adaptive image receive coil.

**Table 3:** Subjective image quality of ZTE-CAA and ZTE-AIR by two independent readers (*n* = 66).

	Reader 1				Reader 2			
	ZTE-CAA	ZTE-AIR	*p*-Value	Effect Size	ZTE-CAA	ZTE-AIR	*p*-Value	Effect Size
**Vessels**	4.74 ± 0.56	4.89 ± 0.31	0.003	0.063	4.73 ± 0.54	4.83 ± 0.41	0.007	0.053
**Bronchi**	3.59 ± 0.68	3.98 ± 0.64	<0.001	0.183	3.52 ± 0.64	4.82 ± 0.70	<0.001	0.156
**Fissures**	2.45 ± 1.13	2.79 ± 1.00	<0.001	0.130	2.53 ± 1.03	2.77 ± 0.87	<0.001	0.137
**Noise/Artifacts**	3.36 ± 1.11	3.89 ± 0.86	<0.001	0.153	3.14 ± 1.12	3.50 ± 1.04	<0.001	0.175
**Overall Acceptability**	3.5 ± 1.04	3.89 ± 0.88	<0.001	0.128	3.29 ± 1.12	3.83 ± 0.92	<0.001	0.125

Data are presented as mean and standard deviation. A *p*-value < 0.05 was considered statistically significant. ZTE-CAA, zero echo time lung MR imaging using conventional anterior array coil; ZTE-AIR, zero echo time lung MR imaging using adaptive image receive coil.

**Table 4:** Comparison of diagnostic performance for nodules and emphysema on a zone-by-zone basis between ZTE-CAA and ZTE-AIR.

Reader 1				Reader 2		
	ZTE-CAA	ZTE-AIR	*p*-Value	ZTE-CAA	ZTE-AIR	*p*-Value
Nodules						
**Sensitivity**	87.0 (47/54)	88.9 (48/54)	1.00	85.2 (46/54)	88.9 (48/54)	0.625
**Specificity**	98.1 (206/210)	98.6 (207/210)	1.00	98.1 (206/210)	98.6 (207/210)	1.00
**Accuracy**	95.8 (253/264)	96.6 (255/264)	0.625	95.5 (252/264)	96.6 (255/264)	0.375
Emphysema/cysts						
**Sensitivity**	78.3 (36/46)	91.3 (42/46)	0.031	73.9 (34/46)	89.1 (41/46)	0.039
**Specificity**	98.6 (215/218)	98.6 (215/218)	1.00	98.6 (215/218)	99.5 (217/218)	0.500
**Accuracy**	95.1 (251/264)	97.3 (257/264)	0.031	94.3 (249/264)	97.7 (258/264)	0.012

Numbers are shown in percentages. Raw data are shown in parentheses. A *p*-value < 0.05 was considered statistically significant. ZTE-CAA, zero echo time lung MR imaging using conventional anterior array coil; ZTE-AIR, zero echo time lung MR imaging using adaptive image receive coil.

**Table 5:** Comparison of sensitivity on nodule-wise evaluation between ZTE-CAA and ZTE-AIR.

	Reader 1			Reader 2		
	ZTE-CAA	ZTE-AIR	*p*-Value	ZTE-CAA	ZTE-AIR	*p*-Value
**All**	80.0 (64/80)	88.8 (71/80)	0.065	80.0 (64/80)	87.5 (70/80)	0.070
**1–3 cm**	93.1 (27/29)	93.1 (27/29)	1.000	96.8 (28/29)	93.1 (27/29)	1.000
**<1 cm**	72.5 (37/51)	86.3 (44/51)	0.039	70.6 (36/51)	84.3 (43/51)	0.016

Numbers are shown in percentages. Raw data are shown in parentheses. A *p*-value < 0.05 was considered statistically significant. ZTE-CAA, zero echo time lung MR imaging using conventional anterior array coil; ZTE-AIR, zero echo time lung MR imaging using adaptive image receive coil.

## Data Availability

The datasets generated or analyzed during the study are available from the corresponding author on reasonable request.

## Supplementary Materials

The following are available online at https://www.mdpi.com/article/10.3390/diagnostics12010148/s1, Figure S1: Measurement of image sharpness, Table S1: Interobserver agreements in the evaluation of intrapulmonary structures and lesions in two ZTE datasets, Table S2: Patients’ response regarding comfort level.
