# Supplementary material for: Application of Highly Flexible Adaptive Image Receive Coil for Lung MR Imaging Using Zero TE Sequence: Comparison with Conventional Anterior Array Coil
Source: Diagnostics (Basel). 2022 Jan 8;12(1):148. doi: 10.3390/diagnostics12010148 (PMC8774338; doi:10.3390/diagnostics12010148)
Supplement: Supplementary file 1 [file diagnostics-12-00148-s001.zip › diagnostics-1516384-supplementary.pdf]

Supplementary material

Article: Application of Highly Flexible Adaptive Image Receive Coil for Lung MR Imaging using Zero TE Sequence: Comparison with Conventional Anterior Array Coil

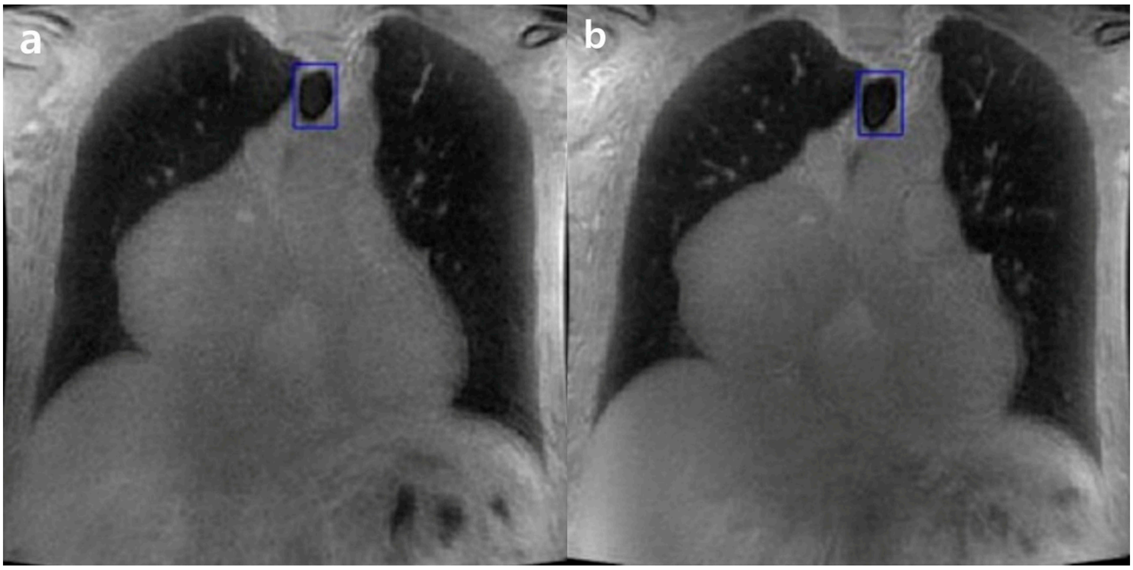

Figure S1. To measure image sharpness, rectangular region of interest was drawn on trachea in original coronal ZTE images using conventional anterior array coil (ZTE-CAA) (a) and Adaptive Image Receive coil (ZTE-AIR) (b), respectively.

Table S1. Interobserver agreements in the evaluation of intrapulmonary structures and lesions in two ZTE datasets.

Structures/ lesions	Weighted kappa	
	ZTE-AIR	ZTE-CAA
Vessels	0.748	0.824
Bronchi	0.588	0.423
Noise/ artifacts	0.773	0.779
Overall acceptability	0.775	0.756
Nodule	0.75	0.616
Emphysema/ and or lung cysts	0.685	0.7

ZTE-AIR, zero echo time lung MR image using Adaptive Image Receive coil; ZTE-CAA, zero echo time lung MR image using conventional anterior array coil

Table S2. Patients' response regarding comfort level

Score	No. of patients
1 (much more comfortable with CAA)	0
2 (slightly more comfortable with CAA)	0
3 (equally comfortable)	2 (3)
4 (slightly more comfortable with AIR)	26 (39.4)
5 (much more comfortable with AIR)	38 (57.6)
Total	66 (100)

Numbers in parentheses are percentages. CAA, conventional anterior array coil; AIR, Adaptive Image Receive coil.
